# Supplementary material for: Distinct immune responses in people living with HIV following SARS-CoV-2 recovery
Source: Commun Med (Lond). 2025 Apr 23;5:132. doi: 10.1038/s43856-025-00839-1 (PMC12018938; doi:10.1038/s43856-025-00839-1)
Supplement: Supplementary file 2 — Supplementary Information [file 43856_2025_839_MOESM2_ESM.pdf]

Supplementary Information

Supplementary Tables

Supplementary Table 1. Association of HIV status with binding Ab stratified by COVID-19 severity, adjusting for SARS-CoV-2 infection severity, age, sex assigned at birth, currently smoking cigarettes/marijuana, region and days since SARS-CoV-2 diagnosis at the enrollment (endemic antigens)

Isotype	Antigen	Comparison	GMR	95% CI	P-value	Q-value
IgG1	229E-RBD	HIV+ vs HIV- in Asymptomatic	1.55	[0.66, 3.66]	0.314	0.376
		HIV+ vs HIV- in Symptomatic	1.2	[0.62, 2.3]	0.593	0.593
		HIV+ vs HIV- in Hospitalized	1.62	[0.88, 2.98]	0.122	0.197
	HKU1-RBD	HIV+ vs HIV- in Asymptomatic	2.11	[0.92, 4.82]	0.078	0.156
		HIV+ vs HIV- in Symptomatic	1.96	[1.04, 3.69]	0.037	0.11
		HIV+ vs HIV- in Hospitalized	1.57	[0.87, 2.84]	0.131	0.197
	NL63-RBD	HIV+ vs HIV- in Asymptomatic	1.56	[0.73, 3.32]	0.25	0.333
		HIV+ vs HIV- in Symptomatic	**1.77**	**[1, 3.16]**	**0.052**	**0.124**
		HIV+ vs HIV- in Hospitalized	**2.14**	**[1.25, 3.68]**	**0.006**	**0.033**
	OC43-RBD	HIV+ vs HIV- in Asymptomatic	1.57	[0.6, 4.12]	0.364	0.397
		HIV+ vs HIV- in Symptomatic	**2.84**	**[1.36, 5.94]**	**0.006**	**0.033**
		HIV+ vs HIV- in Hospitalized	**2.39**	**[1.18, 4.83]**	**0.016**	**0.063**
IgG3	229E-RBD	HIV+ vs HIV- in Asymptomatic	0.58	[0.24, 1.39]	0.224	0.536
		HIV+ vs HIV- in Symptomatic	1.12	[0.58, 2.19]	0.735	0.802
		HIV+ vs HIV- in Hospitalized	**2.3**	**[1.23, 4.28]**	**0.009**	**0.052**
	HKU1-RBD	HIV+ vs HIV- in Asymptomatic	0.67	[0.3, 1.49]	0.329	0.564
		HIV+ vs HIV- in Symptomatic	1.03	[0.56, 1.88]	0.935	0.935
		HIV+ vs HIV- in Hospitalized	1.94	[1.1, 3.42]	0.022	0.087
	NL63-RBD	HIV+ vs HIV- in Asymptomatic	0.49	[0.21, 1.15]	0.103	0.308
		HIV+ vs HIV- in Symptomatic	1.41	[0.74, 2.71]	0.299	0.564
		HIV+ vs HIV- in Hospitalized	1.19	[0.65, 2.18]	0.582	0.723
	OC43-RBD	HIV+ vs HIV- in Asymptomatic	0.78	[0.33, 1.81]	0.563	0.723
		HIV+ vs HIV- in Symptomatic	1.19	[0.62, 2.26]	0.603	0.723
		HIV+ vs HIV- in Hospitalized	**2.54**	**[1.39, 4.63]**	**0.002**	**0.029**
IgA	229E-RBD	HIV+ vs HIV- in Asymptomatic	1.06	[0.56, 2]	0.87	0.87
		HIV+ vs HIV- in Symptomatic	1.24	[0.76, 2.03]	0.381	0.784
		HIV+ vs HIV- in Hospitalized	1.12	[0.71, 1.77]	0.618	0.784
	HKU1-RBD	HIV+ vs HIV- in Asymptomatic	1.4	[0.69, 2.83]	0.348	0.784
		HIV+ vs HIV- in Symptomatic	1.36	[0.8, 2.33]	0.256	0.784
		HIV+ vs HIV- in Hospitalized	1.1	[0.66, 1.81]	0.718	0.784
	NL63-RBD	HIV+ vs HIV- in Asymptomatic	1.14	[0.57, 2.28]	0.711	0.784
		HIV+ vs HIV- in Symptomatic	1.24	[0.73, 2.11]	0.42	0.784
		HIV+ vs HIV- in Hospitalized	0.81	[0.5, 1.33]	0.414	0.784
	OC43-RBD	HIV+ vs HIV- in Asymptomatic	0.76	[0.37, 1.57]	0.464	0.784
		HIV+ vs HIV- in Symptomatic	1.38	[0.8, 2.39]	0.247	0.784
		HIV+ vs HIV- in Hospitalized	1.15	[0.69, 1.92]	0.592	0.784

GMR: Geometric mean ratio
Significant differences (p-values <0.05 and q-values <0.2) are indicated in bold

Supplementary Table 2. Association of HIV status with functional markers stratified by COVID-19 severity, adjusting for SARS-CoV-2 infection severity, age, sex assigned at birth, currently smoking cigarettes/marijuana, region and days since SARS-CoV-2 diagnosis at the enrollment

		Response Rate				Magnitudes among Positive Responders			
**Assay**	**Comparison**	**OR**	**95% CI**	**P-value**	**Q-value**	**GMR**	**95% CI**	**P-value**	**Q-value**
ADCP	HIV+ vs HIV- in Asymptomatic	1.83	[0.3, 10.98]	0.511	0.557	0.82	[0.55, 1.23]	0.336	0.576
	HIV+ vs HIV- in Symptomatic	4.79	[0.24, 94.49]	0.303	0.455	0.86	[0.67, 1.1]	0.226	0.541
	HIV+ vs HIV- in Hospitalized	0.41	[0.1, 1.66]	0.212	0.455	1.22	[0.95, 1.57]	0.115	0.346
I-ADCC	HIV+ vs HIV- in Asymptomatic	2.97	[0.55, 15.92]	0.204	0.455	1.08	[0.59, 2]	0.797	0.869
	HIV+ vs HIV- in Symptomatic	0.47	[0.13, 1.63]	0.231	0.455	0.8	[0.51, 1.25]	0.322	0.576
	HIV+ vs HIV- in Hospitalized	0.45	[0.13, 1.58]	0.211	0.455	0.92	[0.69, 1.22]	0.548	0.822
T-ADCC	HIV+ vs HIV- in Asymptomatic	2.62	[0.48, 14.37]	0.266	0.455	1.07	[0.67, 1.72]	0.766	0.869
	HIV+ vs HIV- in Symptomatic	2.5	[0.66, 9.53]	0.179	0.455	0.77	[0.6, 0.98]	0.037	0.222
	HIV+ vs HIV- in Hospitalized	0.91	[0.26, 3.17]	0.876	0.876	0.97	[0.78, 1.2]	0.761	0.869
SECABA	HIV+ vs HIV- in Asymptomatic	2.01	[0.34, 11.75]	0.44	0.528	1.02	[0.75, 1.38]	0.915	0.915
	HIV+ vs HIV- in Symptomatic	3.78	[0.19, 75.4]	0.383	0.511	0.84	[0.69, 1.02]	0.085	0.339
	HIV+ vs HIV- in Hospitalized	7.17	[0.35, 145.62]	0.2	0.455	**0.79**	**[0.67, 0.95]**	**0.011**	**0.137**

OR: Odds ratio; GMR: Geometric mean ratio

Significant differences (p-values <0.05 and q-values <0.2) are indicated in bold

Supplementary Table 3. Association of HIV status with MSD IgG stratified by COVID-19 severity, adjusting for SARS-CoV-2 infection severity, age, sex assigned at birth, currently smoking cigarettes/marijuana, region and days since SARS-CoV-2 diagnosis at the enrollment

		Response Rate				Magnitudes among Positive Responders			
**Antigen**	**Comparison**	**OR**	**95% CI**	**P-value**	**Q-value**	**GMR**	**95% CI**	**P-value**	**Q-value**
CoV-2 Spike	HIV+ vs HIV- in Asymptomatic	2.28	[0.4, 13.07]	0.355	0.602	1.85	[0.62, 5.53]	0.271	0.406
	HIV+ vs HIV- in Symptomatic	4.21	[0.2, 88.97]	0.355	0.602	**0.43**	**[0.21, 0.9]**	**0.024**	**0.11**
	HIV+ vs HIV- in Hospitalized	1.72	[0.23, 13]	0.602	0.677	1.04	[0.52, 2.09]	0.911	0.911
CoV-2 RBD	HIV+ vs HIV- in Asymptomatic	2.12	[0.37, 12.29]	0.401	0.602	2.25	[0.83, 6.08]	0.112	0.335
	HIV+ vs HIV- in Symptomatic	4.57	[0.23, 92.22]	0.322	0.602	**0.46**	**[0.24, 0.89]**	**0.022**	**0.11**
	HIV+ vs HIV- in Hospitalized	2.36	[0.33, 17.16]	0.395	0.602	0.78	[0.42, 1.48]	0.451	0.579
CoV-2 Nucleocapsid	HIV+ vs HIV- in Asymptomatic	1.62	[0.28, 9.26]	0.586	0.677	2.13	[0.69, 6.59]	0.188	0.406
	HIV+ vs HIV- in Symptomatic	4.77	[0.24, 94.99]	0.306	0.602	0.64	[0.3, 1.36]	0.244	0.406
	HIV+ vs HIV- in Hospitalized	1.14	[0.23, 5.8]	0.87	0.87	0.84	[0.4, 1.75]	0.635	0.715

OR: Odds ratio; GMR: Geometric mean ratio

Significant differences (p-values <0.05 and q-values <0.2) are indicated in bold

Supplementary Table 4. Association of HIV status with ACE-2 blocking stratified by COVID-19 severity, adjusting for SARS-CoV-2 infection severity, age, sex assigned at birth, currently smoking cigarettes/marijuana, region and days since SARS-CoV-2 diagnosis at the enrollment

		Response Rate			Magnitudes among Positive Responders		
**Assay**	**Comparison**	**OR**	**95% CI**	**P-value**	**OR**	**95% CI**	**P-value**
ACE-2	HIV+ vs HIV- in Asymptomatic	2.38	[0.4, 14.01]	0.338	2.58	[0.38, 17.61]	0.333
	HIV+ vs HIV- in Symptomatic	0.72	[0.12, 4.52]	0.729	0.37	[0.08, 1.66]	0.197
	HIV+ vs HIV- in Hospitalized	4.24	[0.18, 100.56]	0.371	0.9	[0.3, 2.76]	0.86

OR: Odds ratio

Supplementary Table 5. Association of HIV status with nAb response rate and titer among positive responders stratified by SARS-CoV-2 infection severity, adjusting for COVID-19 severity, age, sex assigned at birth, currently smoking cigarettes/marijuana, region and days since SARS-CoV-2 diagnosis at the enrollment

		Response Rate				Magnitudes among Positive Responders			
**ID50/ID80**	**Comparison**	**OR**	**95% CI**	**P-value**	**Q-value***	**GMR**	**95% CI**	**P-value**	**Q-value**
ID50	HIV+ vs HIV- in Asymptomatic	2.91	[0.52, 16.45]	0.226	0.34	1.77	[0.72, 4.32]	0.21	0.315
	HIV+ vs HIV- in Symptomatic	7.17	[0.35, 145.61]	0.2	0.34	**0.53**	**[0.29, 0.96]**	**0.037**	**0.112**
	HIV+ vs HIV- in Hospitalized	1.22	[0.23, 6.31]	0.816	0.816	0.86	[0.48, 1.52]	0.599	0.599
ID80	HIV+ vs HIV- in Asymptomatic	2.91	[0.52, 16.45]	0.226	0.34	1.99	[0.86, 4.57]	0.106	0.212
	HIV+ vs HIV- in Symptomatic	7.17	[0.35, 145.61]	0.2	0.34	**0.54**	**[0.31, 0.94]**	**0.029**	**0.112**
	HIV+ vs HIV- in Hospitalized	1.22	[0.23, 6.31]	0.816	0.816	0.79	[0.46, 1.36]	0.401	0.482

OR: Odds ratio; GMR: Geometric mean ratio

Significant differences (p-values <0.05 and q-values <0.2) are indicated in bold

Supplementary Table 6. Association of HIV status with B cells subset frequency stratified by COVID-19 severity, adjusting for SARS-CoV-2 infection severity, age, sex assigned at birth, currently smoking cigarettes/marijuana, region and days since SARS-CoV-2 diagnosis at the enrollment

B-cell	Comparison	GMR	95% CI	P-value	Q-value
%RBD+ IgA+ of Memory B cells	HIV+ vs HIV- in Asymptomatic	1.33	[0.35, 5.02]	0.675	0.985
	HIV+ vs HIV- in Symptomatic	0.76	[0.27, 2.09]	0.59	0.985
	HIV+ vs HIV- in Hospitalized	1.35	[0.52, 3.48]	0.533	0.985
%RBD+ IgG+ of Memory B cells	HIV+ vs HIV- in Asymptomatic	1.16	[0.28, 4.81]	0.837	0.985
	HIV+ vs HIV- in Symptomatic	1.25	[0.42, 3.71]	0.681	0.985
	HIV+ vs HIV- in Hospitalized	1.1	[0.4, 3.03]	0.853	0.985
%RBD+ IgM+ of Memory B cells	HIV+ vs HIV- in Asymptomatic	1.34	[0.41, 4.39]	0.627	0.985
	HIV+ vs HIV- in Symptomatic	0.83	[0.34, 2.05]	0.687	0.985
	HIV+ vs HIV- in Hospitalized	1.17	[0.5, 2.72]	0.719	0.985
%RBD+ of %S+ IgG+ B cells	HIV+ vs HIV- in Asymptomatic	1.55	[0.12, 20.41]	0.739	0.985
	HIV+ vs HIV- in Symptomatic	3.95	[0.61, 25.61]	0.15	0.985
	HIV+ vs HIV- in Hospitalized	0.96	[0.17, 5.56]	0.966	0.985
%RBD+ of total B cells	HIV+ vs HIV- in Asymptomatic	1.28	[0.59, 2.79]	0.538	0.985
	HIV+ vs HIV- in Symptomatic	1.21	[0.67, 2.2]	0.529	0.985
	HIV+ vs HIV- in Hospitalized	1.21	[0.69, 2.11]	0.5	0.985
%S+ IgA+ of Memory B cells	HIV+ vs HIV- in Asymptomatic	0.87	[0.27, 2.8]	0.811	0.985
	HIV+ vs HIV- in Symptomatic	1.16	[0.47, 2.83]	0.749	0.985
	HIV+ vs HIV- in Hospitalized	1.16	[0.5, 2.67]	0.732	0.985
%S+ IgA+ of total B cells	HIV+ vs HIV- in Asymptomatic	1.03	[0.44, 2.37]	0.95	0.985
	HIV+ vs HIV- in Symptomatic	1.07	[0.57, 2.03]	0.829	0.985
	HIV+ vs HIV- in Hospitalized	1.04	[0.57, 1.88]	0.908	0.985
%S+ IgG+ of Memory B cells	HIV+ vs HIV- in Asymptomatic	0.99	[0.34, 2.87]	0.985	0.985
	HIV+ vs HIV- in Symptomatic	0.83	[0.37, 1.86]	0.644	0.985
	HIV+ vs HIV- in Hospitalized	1.14	[0.53, 2.43]	0.738	0.985
%S+ IgG+ of total B cells	HIV+ vs HIV- in Asymptomatic	1.29	[0.5, 3.31]	0.594	0.985
	HIV+ vs HIV- in Symptomatic	0.98	[0.48, 2.01]	0.952	0.985
	HIV+ vs HIV- in Hospitalized	1.04	[0.53, 2.03]	0.919	0.985
%S+ IgM+ IgD+ of total B cells	HIV+ vs HIV- in Asymptomatic	0.98	[0.64, 1.51]	0.941	0.985
	HIV+ vs HIV- in Symptomatic	0.95	[0.69, 1.32]	0.765	0.985
	HIV+ vs HIV- in Hospitalized	0.94	[0.69, 1.28]	0.692	0.985
%S+ IgM+ of Memory B cells	HIV+ vs HIV- in Asymptomatic	1.65	[0.51, 5.36]	0.408	0.985
	HIV+ vs HIV- in Symptomatic	0.68	[0.28, 1.67]	0.396	0.985
	HIV+ vs HIV- in Hospitalized	0.89	[0.39, 2.07]	0.793	0.985
%S+ IgM+ of total B cells	HIV+ vs HIV- in Asymptomatic	1.56	[0.69, 3.5]	0.286	0.985
	HIV+ vs HIV- in Symptomatic	0.77	[0.42, 1.44]	0.418	0.985
	HIV+ vs HIV- in Hospitalized	0.9	[0.51, 1.61]	0.731	0.985
%S+ of total B cells	HIV+ vs HIV- in Asymptomatic	1.12	[0.67, 1.86]	0.665	0.985
	HIV+ vs HIV- in Symptomatic	0.89	[0.6, 1.31]	0.554	0.985
	HIV+ vs HIV- in Hospitalized	0.95	[0.66, 1.37]	0.798	0.985

GMR: Geometric mean ratio; *: multiple-comparison adjustment across all B-cell frequencies;

Supplementary Table 7. Down-selected immune markers after the first selection step further selected for the final classification of PLWH vs PWOH among all participants.

	Selected markers from RFE two steps	No. of times selected in RFE step 2	Selection frequency (%)	Selected markers (*) from CV-SL backward selection	A smaller set of markers (*) with a similar CV-AUC
1	%S+ IgG+ of memory B cells	200	100	*	*
2	%S+ IgG+ of total B cells	200	100		
3	IgG1 to NL63 RBD	200	100	*	*
4	IgG1 to OC43 RBD	200	100	*	
5	ADCP	199	99.5		
6	IgG1 to SARS-CoV-2 NP	199	99.5		
7	%S+ of total B cells	199	99.5		
8	SECABA	199	99.5	*	
9	CD4 ORF3 ICS	198	99		
10	IgA to SC-2 RBD	197	98.5		
11	CD8 Spike ICS	197	98.5	*	*
12	IgG1 to RBD	195	97.5		
13	IgG3 to OC43 RBD	195	97.5	*	*
14	IgA to S6P	193	96.5	*	
15	IgG1 to HKU1 RBD	193	96.5		
16	ACE2 blocking	192	96	*	
17	IgG3 to SARS-CoV-2 NTD	191	95.5		
18	IgG1 to SARS-CoV-2 S2P	189	94.5	*	*
19	IgG3 to SARS-CoV-2 S2P	189	94.5		
20	IgG3 to HKU1 RBD	188	94	*	*
21	IgG3 to SARS-CoV-2 NP	186	93	*	*
22	%RBD+ IgA+ of memory B cells	180	90	*	
**CV-AUC**				**0.7561**	**0.7538**

Supplementary Table 8. Down-selected immune markers after the first selection step further selected for classification of PLWH vs PWOH among participants recovered from symptomatic outpatient COVID-19

	Selected markers from RFE two steps	No. of times selected in RFE step 2	Selection frequency (%)	Selected markers (*) from CV-SL backward
1	%S+ IgG+ of memory B cells	200	100	*
2	IgG1 to OC43 RBD	200	100	*
3	%S+ IgG+ of total B cells	198	99	
4	MSD IgG to RBD	191	95.5	
5	%S+ of total B cells	190	95	*
6	ACE2	186	93	
7	IgG1 to NL63 RBD	180	90	
**CV-AUC**				**0.8495**

Supplementary Table 9. Down-selected immune markers after the first selection step further selected for classification of PLWH vs PWOH among participants recovered from hospitalization from COVID-19

	Marker	No. of times selected in model	Selection frequency (%)	Selected markers (*) from CV-SL backward
1	IgG1 to NL63 RBD	200	100	*
2	IgG3 to HKU1 RBD	199	99.5	
3	IgG3 to OC43 RBD	199	99.5	*
4	ADCP	193	96.5	
5	IgG1 to OC43 RBD	191	95.5	*
6	IgG1 to SARS-CoV-2 NP	177	88.5	*
7	IgG3 to SARS-CoV-2 RBD	172	86	*
**CV-AUC**				**0.8118**

Supplementary Table 10. Comparisons of Spearman correlations between PLWH vs all PWOH samples and 10,000 subsets subsampled from PWOH samples with an equal sample size as the PLWH samples. The measure is the number (%) of Spearman correlations in PWOH greater than PLWH.

Samples	Statistics	All participants	Asymptomatic	Symptomatic outpatient	Hospitalized
**Observed**		1009 (65.5%)	773 (50.2%)	1072 (69.6%)	808 (52.5%)
**10,000 subsamples from PWOH**	Mean	945 (61.4%)	747 (48.5%)	995 (64.6%)	794 (51.6%)
	Median (IQR)	955 (62%)	754 (49%)	1000 (64.9%)	790 (51.3%)
	Interquartile Range	869-1030 (56.4-66.9%)	690-811 (44.8-52.7%)	915-1077 (59.4-69.9%)	698-887 (45.3-57.6%)
	Range	510-1302 (33.1-84.5%)	434-1001 (28.2-65%)	588-1337 (38.2-86.8%)	460-1248 (29.9-81%)
	**Prob(%PWOH>%PLWH)**	**0.918**	**0.427**	**0.972**	**0.557**

Probability that network statistic for PWOH>PLWH is indicated in bold

Supplementary Table 11. List of antigens used in BAMA assay

Antigen name (short)	Antigen name (full)	Source
RBD	SARS-CoV-2 RDB-His	Institute for Protein Design, Seattle, Washington
2P Spike	2019-nCoV S/293F	Duke Protein Production Facility
6P Spike	SARS-CoV-2 S Hexapro_293F	Duke Protein Production Facility
Nucleoprotein (NP)	SARS-CoV-2 Nucleoprotein His	Cedarlane, Burlington, NC; Cat# REC31812-500
NTD	SARS-CoV-2-NTD-AVI Biotin	NIH/NIAID/VRC, Peter Kwong
HKU1-RBD	HKU1-RBD	UNC School of Medicine
229E-RBD	229E-RBD	UNC School of Medicine
NL63-RBD	NL63-RBD	UNC School of Medicine
OC43-RBD	OC43-RBD	UNC School of Medicine

Supplementary Table 12. List of antibodies used in B cell phenotyping

Antibody	Manufacturer	Clone	Catalog
CD3 BV510	BD Biosciences	HIT3a	564713
CD14 BV510	BD Biosciences	MφP9	563079
CD56 BV510	BD Biosciences	NCAM16.2	563041
CD19 BUV395	BD Biosciences	SJ25-C1	563549
CD20 BUV737	BD Biosciences	2H7	564432
CD21 PE-Cy7	BD Biosciences	B-ly4	561374
CD27 BV605	BioLegend	O323	302830
CD38 BB700	BD Biosciences	HIT2	566445
IgA VioBlue	Miltenyi Biotec	IS11-8E10	130-114-005
IgD BV650	BD Biosciences	IA6-2	740594
IgG BV786	BD Biosciences	G18-145	564230
IgM PE/Dazzle 594	BioLegend	MHM-88	314530
R-Phycoerythrin Streptavidin	Invitrogen	N/A	S21388
AlexaFluor 488 Streptavidin	Invitrogen	N/A	S32354
AlexaFluor 647 Streptavidin	Invitrogen	N/A	S32357
LIVE/DEAD Fixable Aqua Stain	Invitrogen	N/A	L34957
CD19 Biotin (used as a control)	BD Biosciences	HIB19	555411

APC, allophycocyanin; BB, brilliant blue; BUV, brilliant ultraviolet; BV, brilliant violet; Cy, cyanine; FITC, fluorescein isothiocyanate; PE R-phycoerythrin; UViD, Live/Dead fixable ultraviolet dead cell stain

Supplementary Table 13. T cell intracellular cytokine staining flow cytometry panel

Specificity	Fluorochrome	Clone	Vendor	Catalogue
Perforin	FITC	B-D48	BioLegend	353310
IL-5	BB630	TRFK5	BD	Custom
IL-13	BB630	JES10-5A2	BD	Custom
Ki67	BB660	B56	BD	Custom
IL-4	BB700	MP4-25D2	BD	Custom
CRTh2	PE	BM16	BioLegend	350106
CD32	PE-Dazzle594	FUN-2	BioLegend	303218
CXCR3 (CD183)	PE-Cy5	1C6/CXCR3	BD	551128
FOXP3	PE-Cy5.5	PCH101	Invitrogen	35-4776-42
IL-17a	PE-Cy7	BL168	BioLegend	512315
IL-2	APC	MQ1-17H12	BioLegend	500310
Granzyme B	Alexa 700	GB11	BD	560213
CD3	APC-Fire750	UCHT1	BioLegend	300470
TNF	BUV395	MAb11	BD	563996
Viability	UViD	N/A	Invitrogen	65-0863
CD45RA	BUV496	HI100	BD	750258
CD19	BUV563	SJ25C1	BD	612916
CD14	BUV661	MΦP9	BD	741684
CD154	BUV737	TRAP1	BD	748983
CD8	BUV805	SK1	BD	612889
IFNγ	V450	B27	BD	560371
CD4	BV480	SK3	BD	566104
CD16	BV570	3G8	BioLegend	302036
CCR7	BV605	G034H7	BioLegend	353224
CD25	BV650	M-A251	BD	563719
CD64	BV711	10.1	BioLegend	305042
CD56	BV750	5.1H11	BioLegend	362556
CCR6 (CD196)	BV786	11A9	BD	563704

APC, allophycocyanin; BB, brilliant blue; BUV, brilliant ultraviolet; BV, brilliant violet; Cy, cyanine; FITC, fluorescein isothiocyanate; PE R-phycoerythrin; UViD, Live/Dead fixable ultraviolet dead cell stain

Supplementary Figures

Supplementary Figure 1.

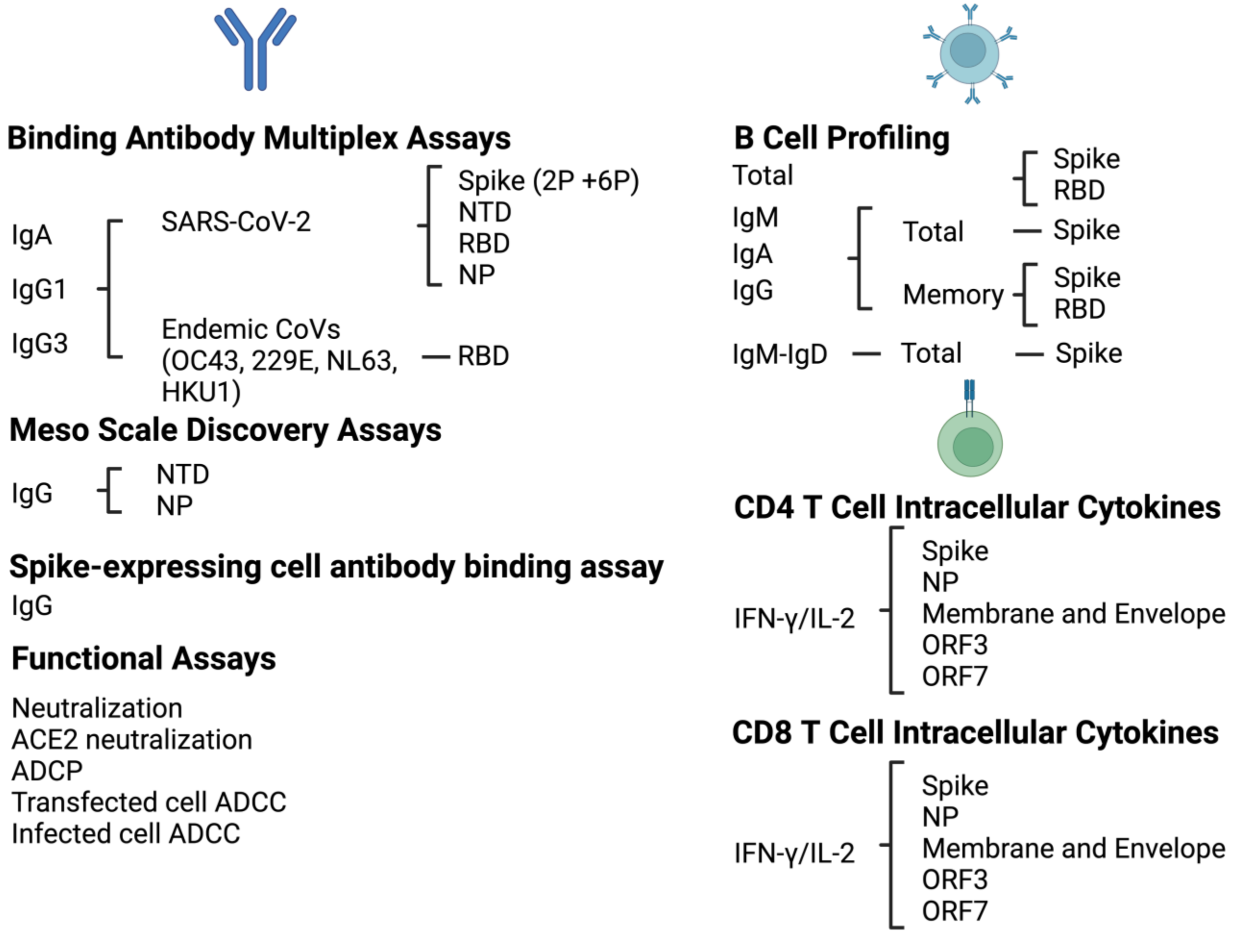

Supplementary Figure 1. Schematic of all assays and immune markers used in the study. Assays measured SARS-CoV-2-specific IgG, IgA, IgG1 and IgG3 binding antibodies, functional antibody responses (neutralizing, ACE2-blocking, ADCC-mediating and ADCP-mediating), IgA+ IgG+ and IgM+ B cells and T cells expressing IFN-γ and/or IL-2.

Supplementary Figure 2.

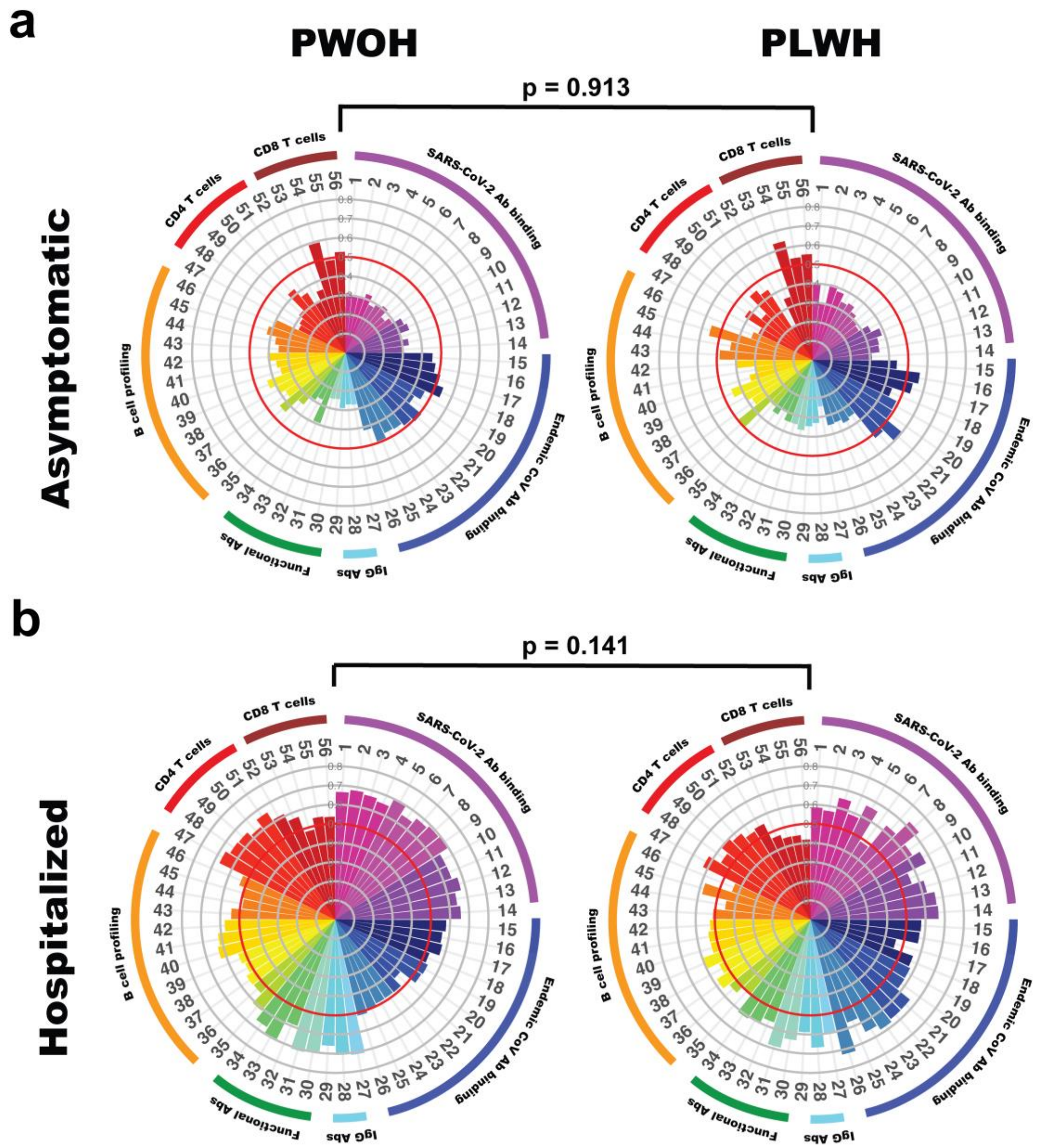

Supplementary Figure 2. No global differences in asymptomatic (A) or hospitalized (B) participants when stratified by HIV status: people without HIV and people living with HIV. Plots indicate the mean quantile for each immune marker and each group. The size of wedge depicts the mean quantile, ranging from 0-1. The red circle indicates the mean quantile (0.5) of all individuals regardless of their HIV status and SARS-CoV-2 disease severity. The p-value indicates the significance of differences in the global distribution of all immune markers between PWOH (n=50 for asymptomatic and 81 for hospitalized) and PLWH (n=9 for asymptomatic and 18 for hospitalized) tested using the direction-projection-permutation test.

Supplementary Figure 3.

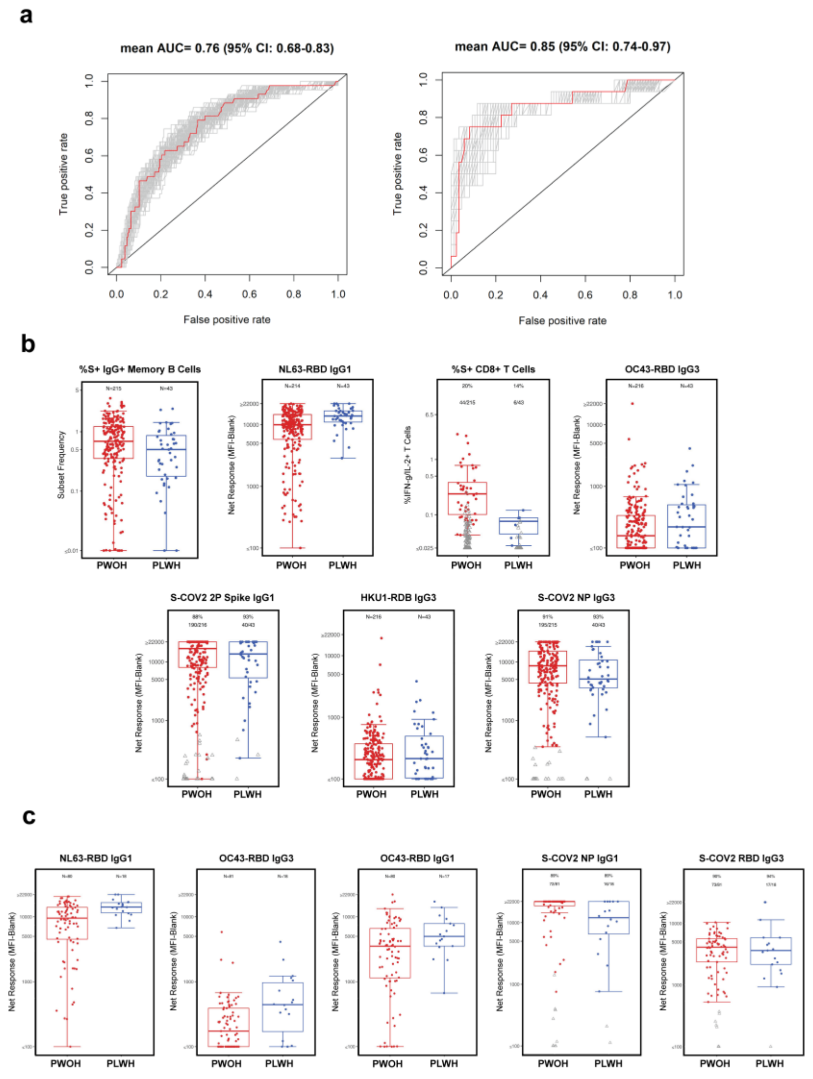

Supplementary Figure 3. Immune markers that predict HIV status regardless of SARS-CoV-2 disease severity and among participants recovered from hospitalization. ROC curves from cross-validated super learning classification of HIV status for all participants (7 markers) and for participants recovered from hospitalization (5 markers) SARS-CoV-2 infection (A). Box plots of immune responses identified as separating PWOH (n=216 for all and 81 for hospitalized) and PLWH (n=43 for all and 18 for hospitalized) recovered from SARS-CoV-2 infection as a whole (B) or those in participants recovered from hospitalization (C). The midline of the box denotes the median and the ends of the box denote the 25th and 75th percentiles among positive responders. The whiskers that extend from the top and bottom of the box extend to the most extreme data points that are no more than 1.5 times the interquartile range (i.e., height of the box) or if no value meets this criterion, to the data extremes. Solid circles represented positive responses and grey triangles represented negative responses. P-values were calculated using the Wilcoxon rank sum test, q-values represent p-values FDR-adjusted for all 56 markers using the Benjamini & Hochberg method.

Supplementary Figure 4.

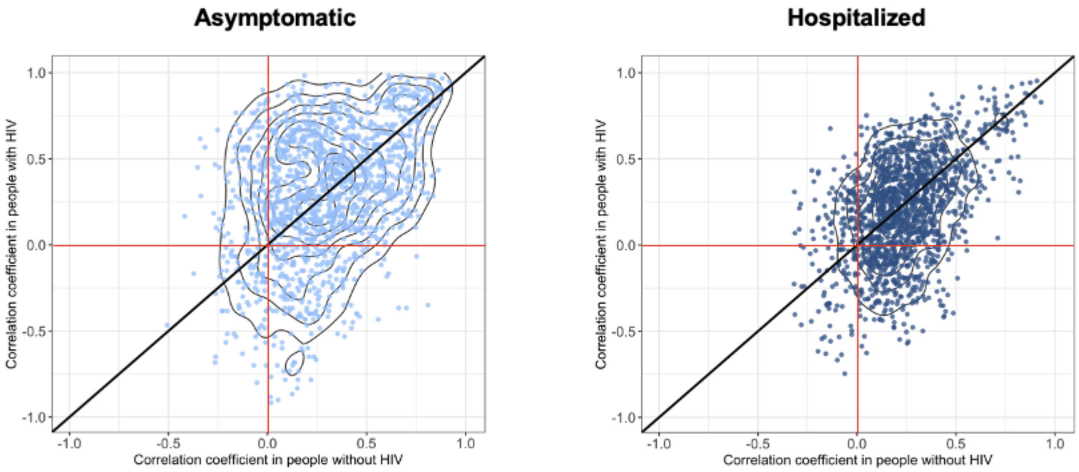

Supplementary Figure 4. Spearman correlations of immune markers stratified by HIV status. Contour plot comparing correlation coefficients in PLWH on the y-axis with correlation coefficients in PWOH on the x-axis among asymptomatic and hospitalized participants. Each point on the contour plot represents a correlation between two immune markers. Lines for Spearman correlation = 0 are shown in red and the identity line (correlation coefficient in PWOH (n=50 for asymptomatic and 81 for hospitalized) is the same as correlation coefficient in PLWH (n=9 for asymptomatic and 18 for hospitalized)) is shown in black.

Supplementary Figure 5.

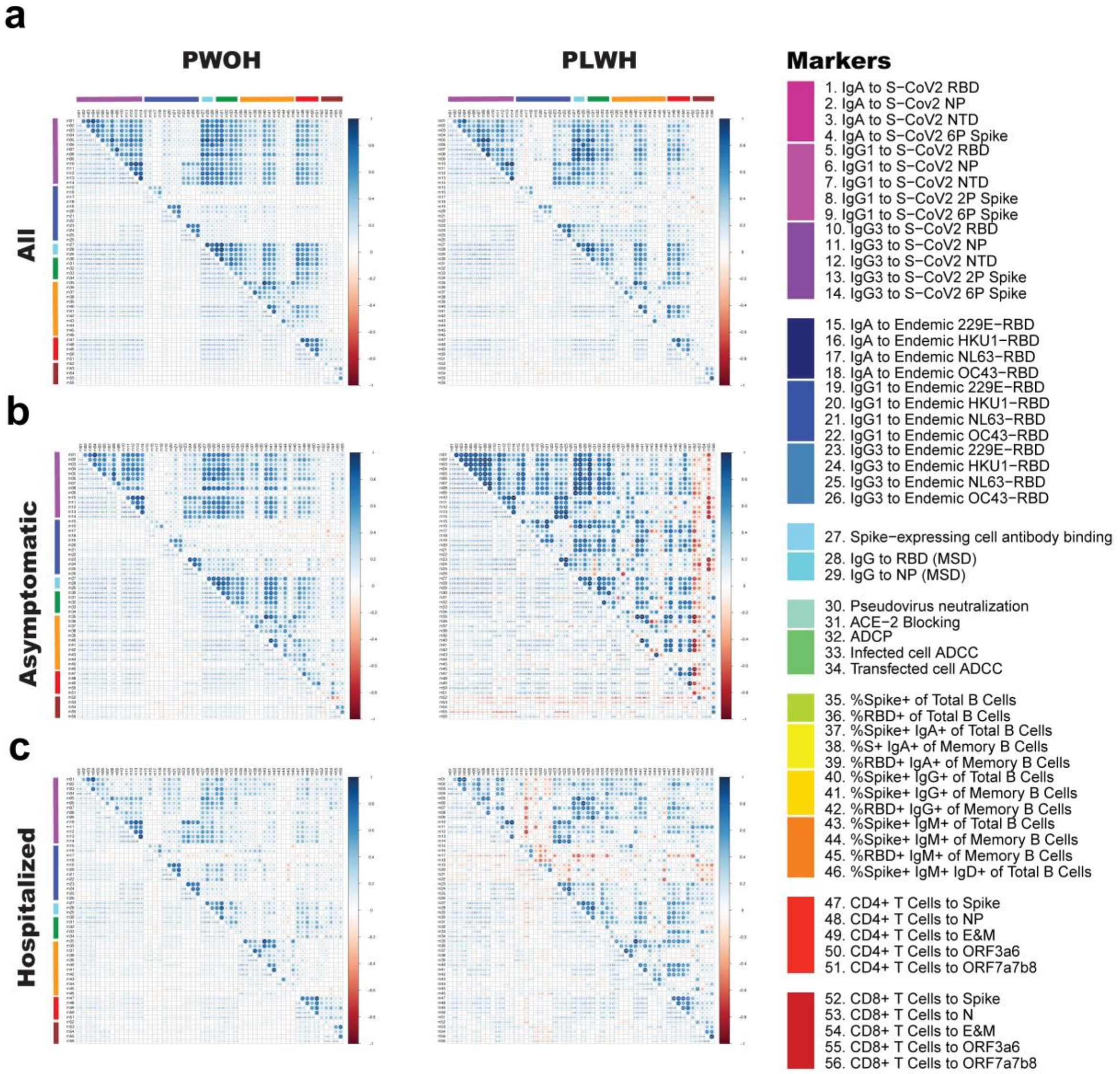

Supplementary Figure 5. Spearman correlations of immune markers stratified by HIV status. Correlation plots of 56 immune markers (44 SARS-CoV-2 and 12 endemic CoV immune markers) among all (A), asymptomatic (B) or hospitalized (C) participants. Spearman correlations are shown by rho values in the lower triangle and circles in the upper triangle colored in blue for positive correlation and red for negative correlation; darker colored and larger sized circles indicating stronger correlations. Significance values are indicated by asterisks in the circles in the upper triangle: * = p-value ≤ 0.05, ** = p-value ≤ 0.01 and *** = p-value ≤ 0.001. The significance of Spearman correlation was tested using the exact two-sided test. All: n=216 for PWOH, n=43 for PLWH, Asymptomatic: n=50 for PWOH, n=9 for PLWH, and Hospitalized: n=81 for PWOH, n=18 for PLWH.

Supplementary Figure 6.

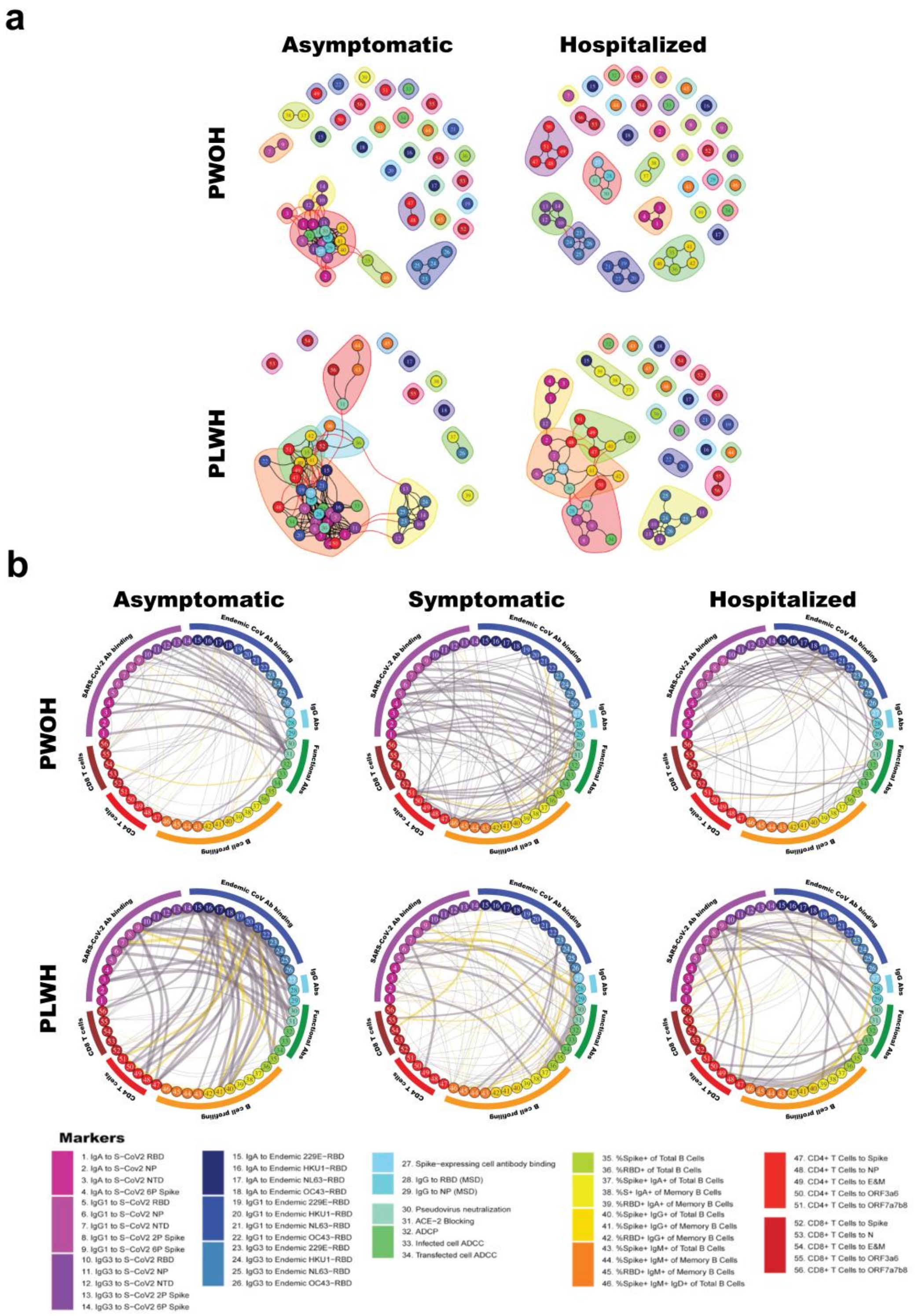

Supplementary Figure 6. Correlation network analyses of 56 immune markers stratified by HIV status and COVID-19 disease severity. Cluster network analysis of immune markers (A). Edges between two markers PWOH or PLWH stratified by SARS-CoV-2 infection severity

indicate an associated correlation coefficient which is significant (FDR<10%) and greater than 0.70 in absolute value based on the exact two-sided test and the Benjamini & Hochberg method. Immune markers are clustered based on edge betweenness. Networks depicting significantly different correlations (B). Each edge connecting two nodes indicates a Spearman's correlation found to be significantly different between people with and without HIV (significance defined as unadjusted p-value<0.05); the color indicates the sign of the correlation: positive in grey and negative in gold. The width of the edges is proportional to the absolute value of the correlation coefficient between the corresponding pair of immune markers. In both types of networks, nodes represent immune markers colored according to marker type. All: n=216 for PWOH, n=43 for PLWH, Asymptomatic: n=50 for PWOH, n=9 for PLWH, Symptomatic outpatient: n=85 for PWOH, n=16 for PLWH, and Hospitalized: n=81 for PWOH, n=18 for PLWH.

Supplementary Figure 7.

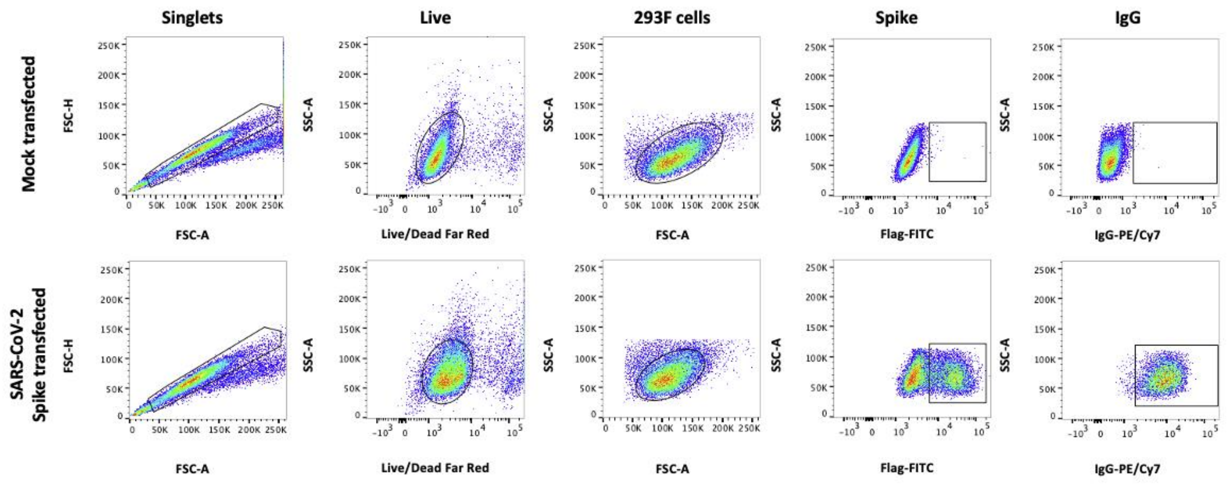

Supplementary Figure 7. Gating strategy for Spike-expressing antibody binding assay. Gates identify singlets, live and intact cells. An anti-flag antibody is used to identify Spike-transfected cells and an anti-IgG Fc antibody to identify Spike+ cells bound by IgG.

Supplementary Figure 8.

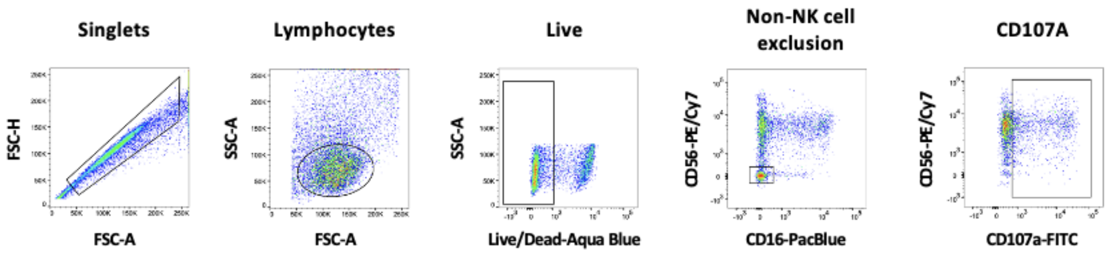

Supplementary Figure 8. Gating strategy for NK cell degranulation assay. Gates identify singlets, lymphocytes and live cells was used to detect cells followed by excluding non-NK cell events and gating for CD107a+ NK cells on mock infected and SARS-CoV-2 Spike-transfected or SARS-CoV-2-infected cells.

Supplementary Figure 9.

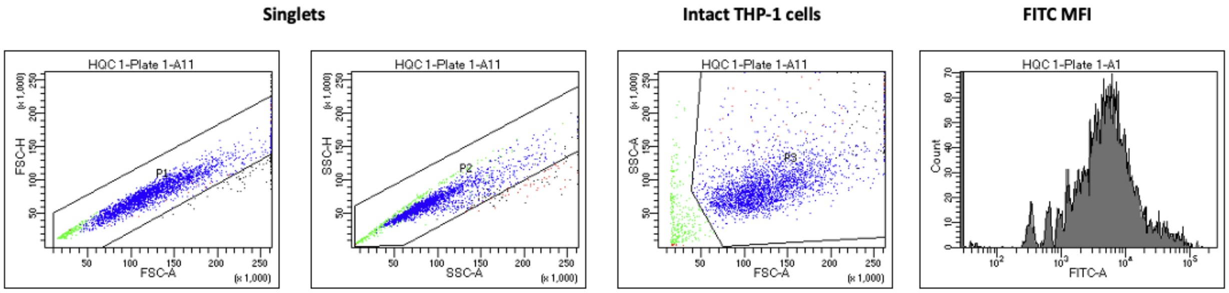

Supplementary Figure 9. Gating strategy for Antibody-dependent phagocytisis assay. Gates identify singlets and intact cells. The mean fluorescent intensity of intact cells is then measured.

Supplementary Figure 10.

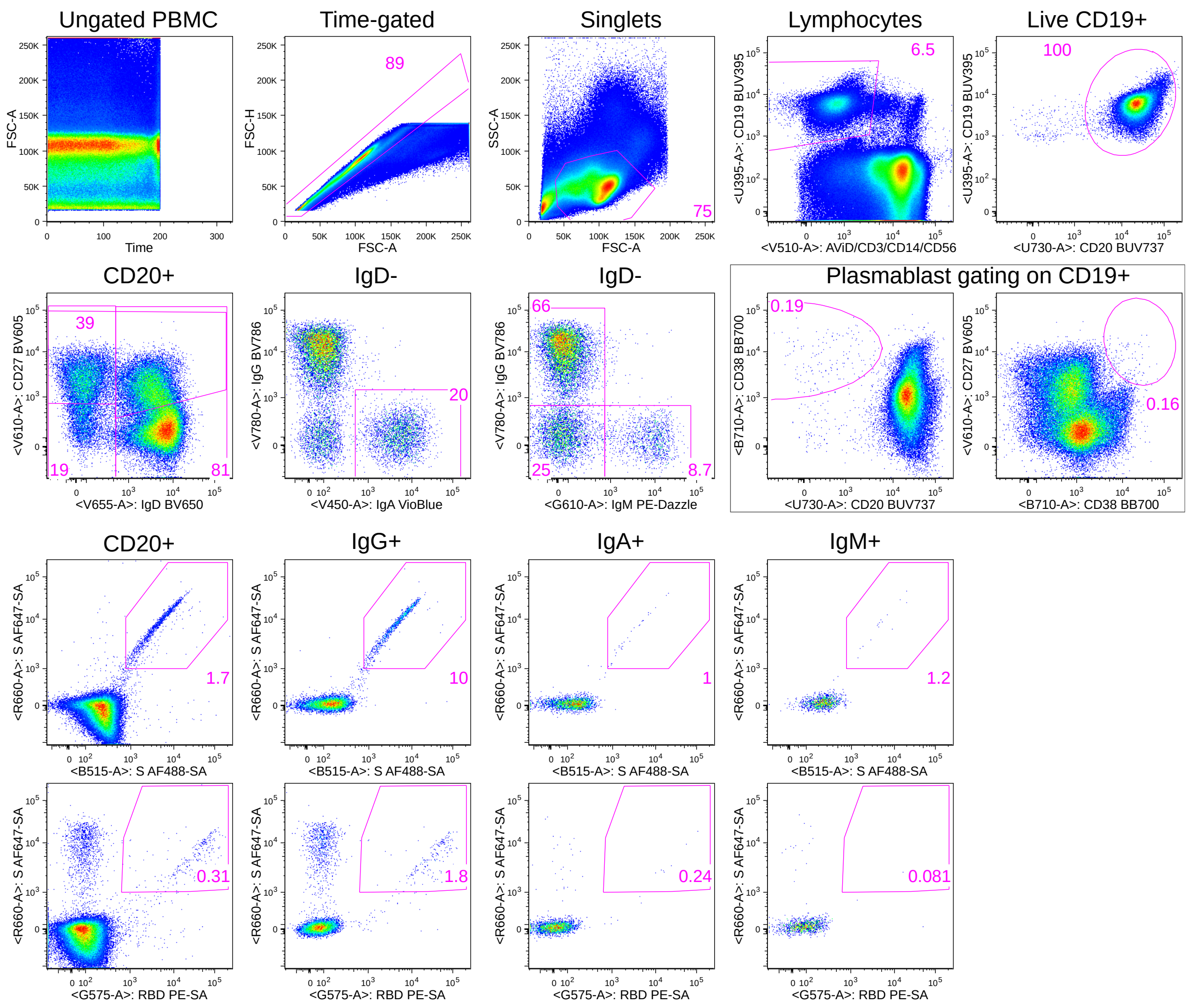

Supplementary Figure 10. Example of the gating strategy of the B cell phenotyping assay. Previously cryopreserved PBMC from a healthy adult donor previously infected with SARS-CoV-2 were stained, and the data were subsequently acquired on a BD FACSymphony instrument. The first row shows the gating hierarchy to identify CD20+ B cells. The second row shows the gates to identify IgD- cells (first plot), IgA+ cells (second plot) and IgG+ and IgM+ cells (third plot). The plots on the right on the second row show two gates to identify plasmablasts. The lower rows show the identification of the spike+ cells by double staining with the spike protein conjugated to AF488 and AF647 (third row) and the identification of the RBD+ cells by staining with the RBD protein conjugated to PE vs. the AF647 spike probe (fourth row). The first column shows the probe+ cells for overall CD20+ cells and the other columns show probe+ for each of the Ig isotypes listed.

Supplementary Figure 11.

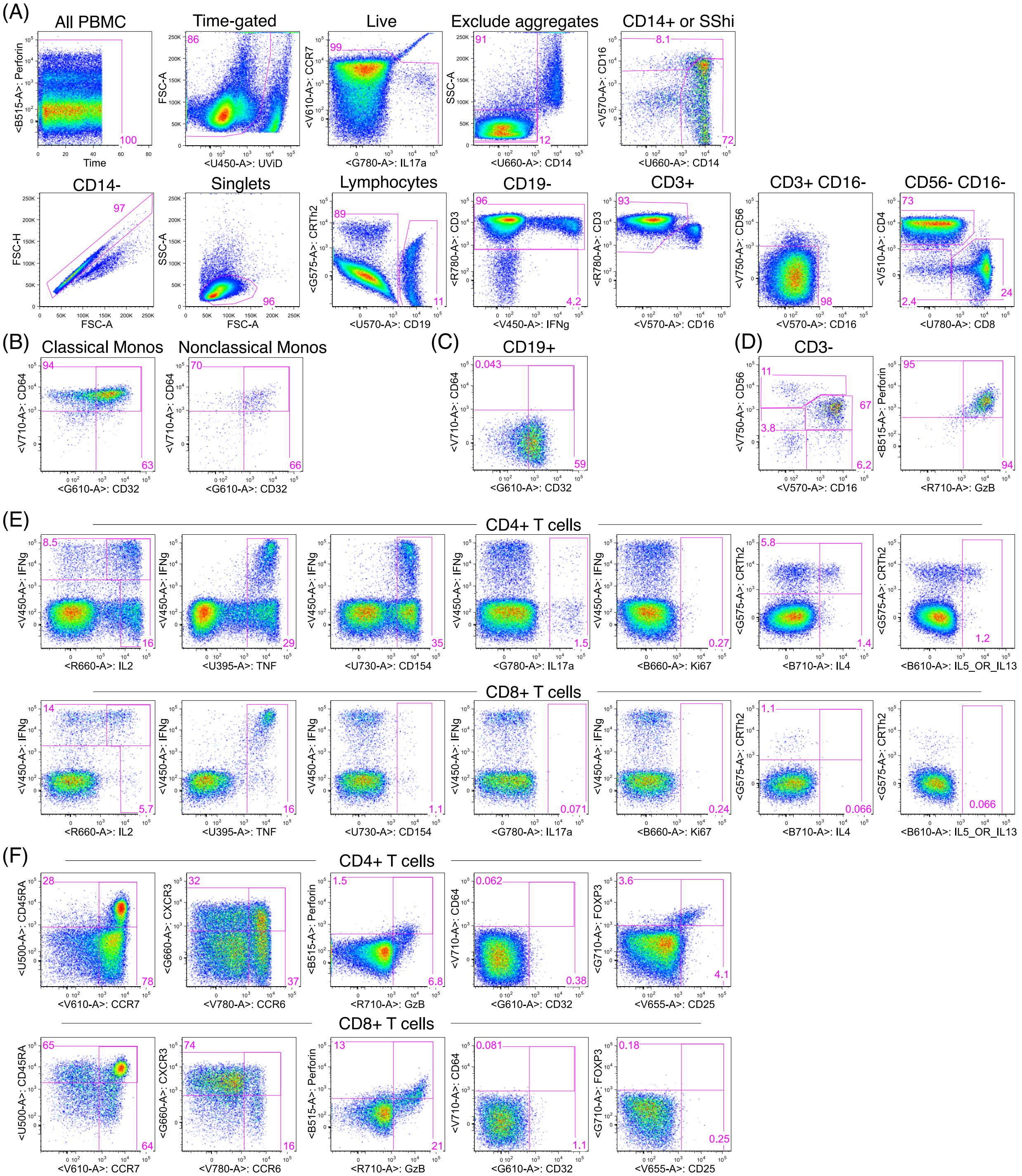

Supplementary Figure 11. Example of the gating strategy of the 27-color ICS assay. Previously cryopreserved PBMC from a healthy adult donor were stimulated with staphylococcal enterotoxin B (SEB) for 6 hours, stained, and the data were subsequently acquired on a BD FACSymphony instrument. (A) Gating hierarchy to identify lineages. Initial gating on time (seconds) to exclude any events early in collection due to pressure fluctuations, live cell gating, exclusion of aggregates (only one plot shown). Monocytes are gated as the inverse of the CD14-

SS^{lo} gate, and the upper right graph shows two monocyte subsets based on CD14 vs. CD16. Non-monocytes are gated as CD14-, followed by singlets, and finally scatter gated on lymphocytes. CD19+ cells are gated against CRTh2 due to the extreme spread of the BUV563 reagent into the G575 detector. CD3+/CD3- cells are gated against IFNg to ensure that any CD3+ cells that have downregulated expression during stimulation are captured. T cells are further defined as CD3+ CD16- on a CD3 vs CD16 plot followed by gating out CD56+ NK T cells on a CD56 vs CD16 plot. Finally, the T cells are further defined by CD4 or CD8 expression on a CD4 vs CD8 plot. (B) CD32 vs. CD64 expression of monocyte subsets. (C) CD32 vs. CD64 expression on CD19+ B cells. (D) NK cell subsets defined by CD16 vs. CD56 or perforin vs. granzyme B on CD3- lymphocytes. (E) Functional markers for CD4+ and CD8+ T cells. A gate is applied for each cytokine and Boolean gates are created to identify cells expressing different combinations of markers. Most gates are copied, applied to all lineages, and cloned so that any changes to the gate on one lineage changes the gate on all lineages. (F) Additional functional and non-functional markers for CD4 and CD8 T cells. As above, these gates are copied and applied to all lineages and cloned, with the exception of CD45RA, due to the difference in expression intensity of this marker on CD8 versus CD4.

For some populations, FMO controls were used to set the lower limits of the gates. Some gates are placed higher to improve the specificity, for example, for the functional markers based on the background as observed in the unstimulated controls.
