# Supplementary material for: Distinct immune responses in people living with HIV following SARS-CoV-2 recovery
Source: Commun Med (Lond). 2025 Apr 23;5:132. doi: 10.1038/s43856-025-00839-1 (PMC12018938; doi:10.1038/s43856-025-00839-1)
Supplement: Supplementary file 3 — Description of Additional Supplementary Files [file 43856_2025_839_MOESM3_ESM.pdf]

Description of Additional Supplementary Files

File name- Supplementary Data 1

File description- Data for the manuscript, a data dictionary and file descriptions (which data were used for generating which figures.

File name- Supplementary Data 2

File description- Association of HIV status with binding Ab stratified by COVID-19 severity, adjusting for SARS-CoV-2 infection severity, age, sex assigned at birth, currently smoking cigarettes/marijuana, region and days since SARS-CoV-2 diagnosis at the enrollment (SARS-CoV-2 antigens)

File name- Supplementary Data 3

File description- Association of HIV status with CD4+/CD8+ T cells stratified by COVID-19 severity, adjusting for SARS-CoV-2 infection severity, age, sex assigned at birth, currently smoking cigarettes/marijuana, region and days since SARS-CoV-2 diagnosis at the enrollment

File name- Supplementary Data 4

File description- CV-AUCs for all combinations of the top seven markers obtained by recursive feature elimination to classify PLWH recovered from symptomatic outpatient infection from PWOH.
