# Supplementary material for: Distinct immune responses in people living with HIV following SARS-CoV-2 recovery
Source: Commun Med (Lond). 2025 Apr 23;5:132. doi: 10.1038/s43856-025-00839-1 (PMC12018938; doi:10.1038/s43856-025-00839-1)
Supplement: Supplementary file 8 — Reporting Summary [file 43856_2025_839_MOESM8_ESM.pdf]

nature portfolio

Corresponding author(s): Dieter Mielke

Last updated by author(s): 12/24/2024

Reporting Summary

Nature Portfolio wishes to improve the reproducibility of the work that we publish. This form provides structure for consistency and transparency in reporting. For further information on Nature Portfolio policies, see our Editorial Policies and the Editorial Policy Checklist.

Statistics

For all statistical analyses, confirm that the following items are present in the figure legend, table legend, main text, or Methods section.

n/a	Confirmed	
☐	☒	The exact sample size (n) for each experimental group/condition, given as a discrete number and unit of measurement
☐	☒	A statement on whether measurements were taken from distinct samples or whether the same sample was measured repeatedly
☐	☒	The statistical test(s) used AND whether they are one- or two-sided<br>*Only common tests should be described solely by name; describe more complex techniques in the Methods section.*
☐	☒	A description of all covariates tested
☐	☒	A description of any assumptions or corrections, such as tests of normality and adjustment for multiple comparisons
☐	☒	A full description of the statistical parameters including central tendency (e.g. means) or other basic estimates (e.g. regression coefficient) AND variation (e.g. standard deviation) or associated estimates of uncertainty (e.g. confidence intervals)
☐	☒	For null hypothesis testing, the test statistic (e.g. F, t, r) with confidence intervals, effect sizes, degrees of freedom and P value noted<br>*Give P values as exact values whenever suitable.*
☒	☐	For Bayesian analysis, information on the choice of priors and Markov chain Monte Carlo settings
☒	☐	For hierarchical and complex designs, identification of the appropriate level for tests and full reporting of outcomes
☐	☒	Estimates of effect sizes (e.g. Cohen's d, Pearson's r), indicating how they were calculated

Our web collection on statistics for biologists contains articles on many of the points above.

Software and code

Policy information about availability of computer code

Data collection	BD FacsDiva, FlowJo v10, Graphpad Prism v9.6
Data analysis	Flow cytometry analyseswere performed using FlowJo v10 and immune profiling analyses using R statistical software.

For manuscripts utilizing custom algorithms or software that are central to the research but not yet described in published literature, software must be made available to editors and reviewers. We strongly encourage code deposition in a community repository (e.g. GitHub). See the Nature Portfolio guidelines for submitting code & software for further information.

Data

Policy information about availability of data

All manuscripts must include a data availability statement. This statement should provide the following information, where applicable:

- Accession codes, unique identifiers, or web links for publicly available datasets
- A description of any restrictions on data availability
- For clinical datasets or third party data, please ensure that the statement adheres to our policy

All data used for analyses in this manuscript will be made publicly available on the public facing HVTN website (https://atlas.scharp.org) upon acceptance of the manuscript

Human research participants

Policy information about studies involving human research participants and Sex and Gender in Research.

Reporting on sex and gender	Sex assigned at birth was collected through self-report. All analyses were performed for aggregated sexes. No separate sex analysis was performed due to a small sample size for female assigned at birth in PLWH. Sex assigned at birth was adjusted as a covariate in the association analysis.
Population characteristics	The study participants characteristics were described in the result section and Table 1.
Recruitment	The samples used in this comprehensive analysis of immune responses were from the subset of participants who were recruited in the HVTN 405/HPTN 1901 trial: a US- and Peru-based observational conducted early in the COVID-19 pandemic. The subset contains all of 43 participants living with HIV (PLWH) recruited in the study and 216 participants without HIV (PWOH) selected to match key demographic characteristics of the PLWH group. This has been described in the result section.
Ethics oversight	IRB approval was granted by a central IRB (Advarra IRB) and, as applicable, by individual clinic sites' IRBs.

Note that full information on the approval of the study protocol must also be provided in the manuscript.

Field-specific reporting

Please select the one below that is the best fit for your research. If you are not sure, read the appropriate sections before making your selection.

☒ Life sciences ☐ Behavioural & social sciences ☐ Ecological, evolutionary & environmental sciences

For a reference copy of the document with all sections, see nature.com/documents/nr-reporting-summary-flat.pdf

Life sciences study design

All studies must disclose on these points even when the disclosure is negative.

Sample size	The sample size was determined by the number of PLWH (n=43) recruited in the parent study HVTN 405/HPTN 1901. About 5:1 ratio of PWOH vs PLWH were selected to match key demographic characteristics of the PLWH group.
Data exclusions	No data were excluded from the analyses. For some sample with missing data for individual assays, we imputed the data using Lasso linear regression method and the mice R package, which was described in the statistical method section.
Replication	All assays were conducted in compliance with Good Clinical Laboratory Practice guidelines for consistency and reproducibility.
Randomization	The samples used in this study were selected from an observational study.
Blinding	All laboratories were blinded to HIV status until after analyses were complete.

Reporting for specific materials, systems and methods

We require information from authors about some types of materials, experimental systems and methods used in many studies. Here, indicate whether each material, system or method listed is relevant to your study. If you are not sure if a list item applies to your research, read the appropriate section before selecting a response.

Materials & experimental systems

n/a	Involved in the study	
☐	☒	Antibodies
☐	☒	Eukaryotic cell lines
☒	☐	Palaeontology and archaeology
☒	☐	Animals and other organisms
☐	☒	Clinical data
☒	☐	Dual use research of concern

Methods

n/a	Involved in the study	
☒	☐	ChIP-seq
☐	☒	Flow cytometry
☒	☐	MRI-based neuroimaging

Antibodies

Antibodies used	All antibodies and their manufacturers and catalog numbers are described in the methods or supplementary tables.

Antibodies used	Binding antibody multiplex assays: mouse anti-human IgG1 (Invitrogen; clone 12G8G11), mouse anti-human IgG3 (Invitrogen; clone HP7047), goat anti-mouse IgG-PE (SouthernBiotech; cat no. 1030-09), goat anti-human IgA-PE (Jackson ImmunoResearch; cat no. 109-006-011).<br>Spike protein-expressing cell antibody binding assay: anti-human IgG-PE/Cy7 (Biolegend; clone HP6017), anti-flag-FITC (Sigma, clone M2).<br>Antibody-dependent NK cell degranulation assays: anti-CD107A-FITC (BD Biosciences, clone H4A3), anti-CD56-PE/Cy7 (BD Biosciences, clone NCAM16.2), anti-CD16-PacBlue (BD Biosciences, clone 3G8), anti-CD69-BV785 (Biolegend, clone FN50)<br>B cell phenotyping: anti-CD3-BV510 (BD Biosciecnes, clone HIT3a), anti-CD14-BV510 (BD Biosciences, clone MpP9), anti-CD56-BV510 (BD Biosciences, clone NCAM16.2), anti-CD19-BUV395 (BD Biosciences, clone SJ25-C1), anti-CD20-BUV737 (BD Biosciences, Clone 2H7), anti-CD21-PE-Cy7 (BD Biosciences, Clone B-ly4), anti-CD27-BV605 (Biolegend, Clone O323), anti-CD38-BB700 (BD Biosciences, HIT2), anti-IgA-VioBlue (Miltenyi Biotec, Clone IS11-8E10), anti-IgD-BV650 (BD Biosciences, clone IA6-2), anti-IgM-PE/Dazzle594 (Biolegend, Clone MHM-88), anti-CD19-Biotin (BD Biosciences, Clone HIB19).<br>Intracellular Cytokine Staining: anti-Perforin-FITC (Biolegend, clone B-D48), anti-IL15-BB630 (BD Biosciences, clone TRFK5), anti-IL-13-BB630 (BD Biosciences, clone JES10-5A2), anti-Ki67-BB660 (BD Biosciences, clone B56), anti-IL-4-BB700 (BD Biosciences, clone MP4-25D2), anti-CRTh2-PE (Biolegend, clone BM16), anti-CD32-PE-Dazzle594 (Biolegend, clone FUN-2), anti-CXCR3-PE-Cy5 (BD Biosciences, clone 1C6/CXCR3), anti-FOXP3-PE-Cy5.5 (Invitrogen, clone PCH101), anti-IL-17a-PE-Cy7 (Biolegend, clone BL168), anti-IL-2-APC (Biolegend, clone MQ1-17H12), anti-Granzyme B-Alexa 700 (BD Biosciences, clone GB11), anti-CD3-APC-Fire750 (Biolegend, clone UCTH1), anti-TNF-BUV395 (BD Biosciences, clone MAb11), anti-CD45RA-BUV496 (BD Biosciences, clone HI100), anti-CD19-BUV563 (BD Biosciences, clone SJ25C1), anti-CD14-BUV661 (BD Biosciences, clone MphiP9), anti-CD154-BUV737 (BD Biosciences, clone TRAP1), anti-CD8-BUV805 (BD Biosciences, clone SK1), anti-IFNg-V450 (BD Biosciences, clone B27), anti-CD4-BV480 (BD BiosBiciences, clone SK3), anti-CD16-BV570 (Biolegend, clone 3G8), anti-CCR7-BV605 (Biolegend, clone G034H7), anti-CD25-BV650 (BD Biosciences, clone M-A251), anti-CD64-BV711 (Biolegend, clone 10.1), anti-CD56-BV750 (Biolegend, clone 5.1H11), anti-CCR6-BV786 (BD osciences, clone 11A9)
Validation	Antibodies are validated by manufacturers and positive and negative controls are described in the text

Eukaryotic cell lines

Policy information about cell lines and Sex and Gender in Research

Cell line source(s)	THP-1 (ATCC); Vero E6 (ATCC); 293T (ATCC); 293F (Thermofisher)
Authentication	Cell lines were authenticated at ATCC or Thermofisher.
Mycoplasma contamination	Cells tested negative for microplasma contamination
Commonly misidentified lines<br>(See ICLAC register)	Not used

Clinical data

Policy information about clinical studies

All manuscripts should comply with the ICMJE guidelines for publication of clinical research and a completed CONSORT checklist must be included with all submissions.

Clinical trial registration	NCT04403880
Study protocol	https://classic.clinicaltrials.gov/ct2/show/NCT04403880
Data collection	Peak symptom severities were self-reported as asymptomatic if no symptoms were present at the time of diagnosis through recovery, symptomatic outpatient if any symptoms were reported but the participant was not hospitalized for COVID-19, and hospitalized if hospitalized due to COVID-19. Detailed information on demographics and comorbitidies were collected at time of enrollment along with self-reported date of positive direct viral detection testing. HIV-1 status, CD4 counts, and HIV-1 viral loads were reported by the enrolling clinics from participants' health records.
Outcomes	A subset of participants enrolled in HVTN405/HPTN1901, comprising of 43 samples from people living with HIV and 216 people without HIV, selected to match key demographic characteristics of the PLWH group, were used.

Flow Cytometry

Plots

Confirm that:

☒ The axis labels state the marker and fluorochrome used (e.g. CD4-FITC).

☒ The axis scales are clearly visible. Include numbers along axes only for bottom left plot of group (a 'group' is an analysis of identical markers).

☒ All plots are contour plots with outliers or pseudocolor plots.

☒ A numerical value for number of cells or percentage (with statistics) is provided.

Methodology

Sample preparation	Sample preparation for each of the relevant assays is described in the Methods section of the manuscript
Instrument	The instrument for each of the relevant assays is described in the Methods section of the manuscript
Software	The software for each of the relevant assays is described in the Methods section of the manuscript
Cell population abundance	Frequencies for populations of interest to the immuno-profiling analyses are described in the manuscript.
Gating strategy	The gating strategy for each of the relevant assays is described in the Methods section of the manuscript

☒ Tick this box to confirm that a figure exemplifying the gating strategy is provided in the Supplementary Information.
